# Effectiveness and Safety of Endovascular Treatment in Large Vessel Occlusion Stroke with an NIHSS Score of ≤5 Exhibiting Predominant Cortical Signs

**DOI:** 10.3390/biomedicines13071700

**Published:** 2025-07-11

**Authors:** Chulho Kim, Seung Joon Oh, Jae Jun Lee, Jong-Hee Sohn, Joo Hye Sung, Yerim Kim, Minwoo Lee, Mi Sun Oh, Kyung-Ho Yu, Hee Jung Mo, Sang-Hwa Lee

**Affiliations:** 1 Department of Neurology, Chuncheon Sacred Heart Hospital, College of Medicine, Hallym University, Chuncheon 24253, Republic of Korea; gumdol52@naver.com (C.K.); deepfoci@hallym.or.kr (J.-H.S.); centertruth@naver.com (J.H.S.); 2 Institute of New Frontier Research Team, Hallym University, Chuncheon 24253, Republic of Korea; scvazd2@naver.com (S.J.O.); iloveu59@hallym.or.kr (J.J.L.); 3 Department of Anesthesiology and Pain, Chuncheon Sacred Heart Hospital, College of Medicine, Hallym University, Chuncheon 24253, Republic of Korea; 4 Department of Neurology, Kangdong Sacred Heart Hospital, College of Medicine, Hallym University, Seoul 05355, Republic of Korea; brainyrk@gmail.com; 5 Department of Neurology, Hallym Sacred Heart Hospital, College of Medicine, Hallym University, Anyang 14068, Republic of Korea; minwoo.lee.md@gmail.com (M.L.); iyyar@hallym.or.kr (M.S.O.); ykh1030@hallym.or.kr (K.-H.Y.); 6 Department of Neurology, Inha University Hospital, Incheon 22332, Republic of Korea; hjungmo@gmail.com

**Keywords:** large vessel occlusion stroke, endovascular treatment, cortical sign, low National Institute of Health Stroke Scale score, three-month modified Rankin scale, bleeding

## Abstract

**Background**: Our study aimed to evaluate the impact of EVT on stroke outcomes in patients with LVO with a National Institute of Health Stroke Scale (NIHSS) score of ≤5, exhibiting primarily cortical signs. **Methods**: We conducted a multicenter registry-based analysis of patients with acute ischemic stroke with LVO who arrived within 12 h of onset. Among these, patients with low NIHSS scores and prominent cortical signs (Items 2, 3, 9, or 11) were included. Patients were divided into two groups: those who underwent EVT and those treated with the best medical therapy (BMT), which included intravenous thrombolysis where appropriate. The primary outcome measure was a modified Rankin scale (mRS) score of 0–1 at 3 months and symptomatic hemorrhagic transformation (SHT). We performed logistic regression analysis to evaluate the impact of EVT on the outcomes. **Results**: Of the 970 patients with LVO, 291 met the inclusion criteria, with 95 and 196 undergoing EVT and BMT, respectively. The EVT group demonstrated a significantly higher rate of 3-month mRS score of 0–1 (65.3% vs. 39.3%, *p* < 0.001) and a lower incidence of SHT than the BMT group (3.2% vs. 12.8%, *p* = 0.01). Multivariate analysis confirmed that EVT was associated with improved functional recovery (mRS score, 0–1; odds ratio [OR], 3.61; 95% confidence interval [CI], 1.82–7.06; *p* < 0.001) and reduced risk of SHT (OR, 0.19; 95% CI, 0.05–0.74; *p* = 0.02). Notably, patients with specific cortical signs, such as aphasia and spatial neglect, exhibited better outcomes with EVT. **Conclusions**: EVT may significantly improve the functional outcomes in patients with mild LVO stroke who present with cortical signs, despite low NIHSS scores. These findings suggest that cortical signs should be a key factor in EVT decision-making for mild stroke cases, thereby advocating for a more individualized approach in acute stroke management.

## 1. Introduction

Current guidelines suggest that endovascular treatment (EVT) should be considered the standard treatment option for acute ischemic stroke with large vessel occlusion (LVO) with an initial National Institute of Health Stroke Scale (NIHSS) score of ≥6 [1]. However, up to 25% of patients with LVO stroke have mild symptoms [2] and 20% of them have worsening symptoms without reperfusion therapy [3,4].

Nevertheless, the benefit of EVT in patients with mild stroke (NIHSS score < 6) remains uncertain because these patients were excluded from pivotal randomized clinical trials (MR CLEAN, ESCAPE, SWIFT PRIME, and EXTEND-IA) [5,6,7]. Recent observational data have raised concerns that EVT could increase the risk of hemorrhagic complications without significantly improving clinical outcomes for patients with mild LVO [8,9,10]. A recent meta-analysis of observational studies demonstrated conflicting results between EVT and best medical treatment (BMT) in these patients [11,12,13]. However, several observational studies demonstrated potential benefits of EVT in patients with LVO stroke with mild symptoms [14,15,16,17].

These conflicting findings highlight the importance of an individualized assessment when considering EVT for mild LVO cases. When making clinical decisions, it is important to carefully balance the likelihood of stroke progression against the anticipated benefits of reperfusion and the potential risks, including hemorrhagic transformation [18]. However, from a clinician’s perspective, the presence of cortical signs in patients with mild symptoms of LVO should be the primary consideration in determining whether EVT is appropriate. In particular, if not treated promptly, cortical signs are associated with a high risk of cognitive deterioration and functional decline months following the stroke occurrence. Therefore, the presence of a predominant cortical sign, even in the presence of mild initial symptoms, is a critical factor in determining whether EVT is indicated.

Using prospectively collected data from a multicenter web-based registry database, we analyzed clinical information in patients with mild LVO acute stroke and cortical signs. We evaluated the impact of EVT on the stroke outcomes in patients with LVO stroke who had predominantly cortical signs despite an NIHSS score of ≤5.

## 2. Materials and Methods

### 2.1. Study Population

Between January 2015 and April 2024, we enrolled patients with acute ischemic stroke from four university-affiliated hospitals using a multicenter, web-based registry. For this analysis, we selected individuals who presented within 12 h of stroke onset and had LVO in either the anterior or posterior circulation. We defined LVO as an occlusion involving the internal carotid artery, the middle cerebral artery (M1 and proximal M2 segments), the posterior cerebral artery, or the basilar artery [19]. Among these patients, we further included those with an initial NIHSS score of 5 or less. We then identified patients exhibiting predominantly cortical symptoms using NIHSS item scores. The following exclusion criteria were applied: (1) a pre-stroke mRS score of 2 or higher, (2) an absence of initial brain CT or MRI within 24 h of onset, (3) an ASPECTS or posterior circulation ASPECTS score below 6, (4) missing data on early neurological deterioration or 3-month mRS outcomes, and (5) a lack of cortical signs upon admission.

### 2.2. Data Collection and Definition of Parameters

We extracted demographic, clinical, laboratory, and outcome-related information from web-based registry systems at the four participating institutions. All NIHSS items were consecutively recorded for each patient included in the registry. Patients were categorized into two groups based on treatment modality: the EVT group (those who received EVT alone or in combination with IVT) and the BMT group (those treated with IVT alone or other standard medical management). For patients with anterior circulation LVO, collateral circulation was assessed using multiphasic CT angiography and classified as good, intermediate, or poor according to the Calgary Stroke Program criteria [20]. In cases of posterior circulation stroke, collateral status was determined using the posterior circulation collateral score (PC-CS) [21]. Two expert vascular neurologists (CK and S-H L), who were blinded to the clinical information, rated the collateral grades independently, yielding high inter-rater reliability (intraclass correlation coefficient [ICC] = 0.92, *p* < 0.001).

### 2.3. Definitions of Cortical Signs

We defined cortical signs as the presence of two or more of the following conditions in this study: aphasia, neglect sign, gaze preference, and visual field defect (VFD). The initial NIHSS was registered by expert neurologists and trained stroke coordinators. We reviewed each item of the initial NIHSS system from the registry database; cortical signs, such as aphasia, were defined as a score of ≥1 for item 9, neglect sign as a score of ≥1 for item 11, gaze preference as a score of ≥1 for item 2, and VFD as a score of ≥1 for item 3 of the NIHSS system in this study.

### 2.4. Outcome Measures

The primary outcome measure was functional outcome defined as a 3-month mRS score of 0–1 and symptomatic hemorrhagic transformation (SHT). The secondary outcomes included early neurological deterioration (END) and an mRS score of 0–2 after three months. END was defined as a worsening of at least one point in the motor component of the NIHSS score or an increase of at least two points in the total NIHSS score within the first 72 h of admission compared to the baseline score [22]. In this study, END was classified into three subtypes based on etiology: stroke progression (e.g., infarct growth, tissue swelling, or surrounding edema), recurrent stroke events, and SHT. Hemorrhagic transformation was defined according to the European Cooperative Acute Stroke Study criteria [23]. Two vascular neurologists (CK and S-HL) reviewed the END data to confirm stroke progression and symptomatic hemorrhagic transformation in a double-blind manner (ICC, 0.88; *p* < 0.001).

### 2.5. Statistical Analysis

We proposed that endovascular treatment (EVT) could increase the likelihood of achieving favorable functional status, defined as a 3-month mRS score of 0–1 after treatment in patients with mild LVO stroke accompanied by cortical signs. Continuous variables were summarized as means with standard deviations, and ordinal variables were expressed as medians with interquartile ranges. Categorical variables were reported as counts and percentages. For group comparisons between the EVT and BMT cohorts, we used Pearson’s chi-square test for categorical data and either a Student’s *t*-test or a Mann–Whitney U test for continuous variables, depending on the distribution. Additionally, we stratified outcomes by individual cortical signs and compared them between groups using chi-square tests.

To determine if EVT influenced outcome measures independently, we conducted binary logistic regression analyses. Variables with *p*-values less than 0.1 in univariate analyses and deemed clinically relevant were included in the multivariable model. We computed both unadjusted and adjusted odds ratios (ORs), along with their 95% confidence intervals (CIs). For the sensitivity analysis, the BMT group was divided into BMT without IVT and IVT only to compare the effects on the stroke outcome of EVT among populations who arrived within 4.5 h of stroke onset through a multivariate analysis. In addition, we compared the impact of EVT on the 3-month mRS scores of 0–1 between the anterior and posterior circulation LVO stroke cases.

## 3. Results

**Figure 1:**
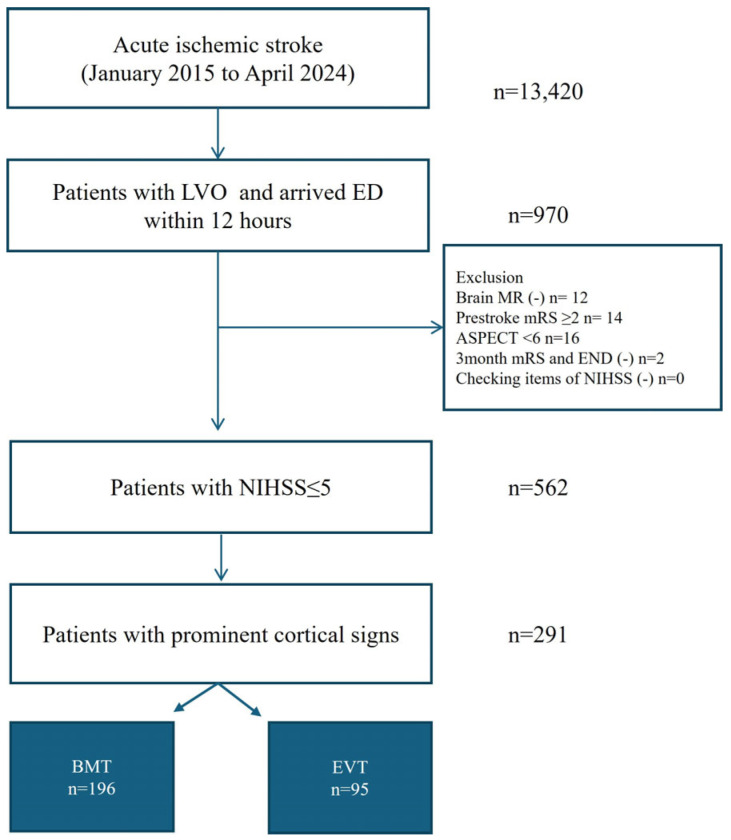
Study flow chart. Abbreviations: LVO—large vessel occlusion; ED—emergency department; MR—magnetic resonance; mRS—modified Rankin Scale; ASPECT—Alberta Stroke Program Early CT score; NIHSS—National Institute of Health Stroke Scale; BMT—best medical treatment; EVT—endovascular treatment.

Among 13,420 consecutive patients with acute ischemic stroke, 970 had LVO and arrived at the hospital within 12 h of stroke onset. Of the 970 patients, 57.9% (n = 562) had initial NIHSS score of ≤5, and 291 with cortical signs who met the inclusion criteria were enrolled in this study (Figure 1); altogether, 67.4% (196/291) and 32.6% (95/291) were enrolled in the BMT and EVT groups, respectively.

In the univariate analysis, most of the variables were not generally significant between the two groups. Collateral status was not different between the BMT and EVT groups. The time interval from the stroke onset to arrival was shorter and the ASPECT score was lower in the EVT group than in the BMT group (Table 1).

**Figure 2:**
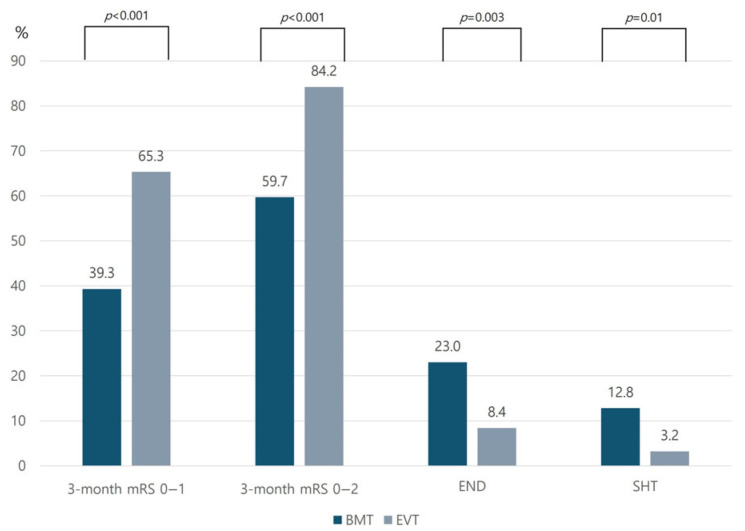
Comparison of stroke outcomes between BMT and EVT groups. Abbreviations: BMT—best medical treatment; EVT—endovascular treatment; mRS—modified Rankin Scale; END—early neurological deterioration; SHT—symptomatic hemorrhagic transformation.

**Figure 3:**
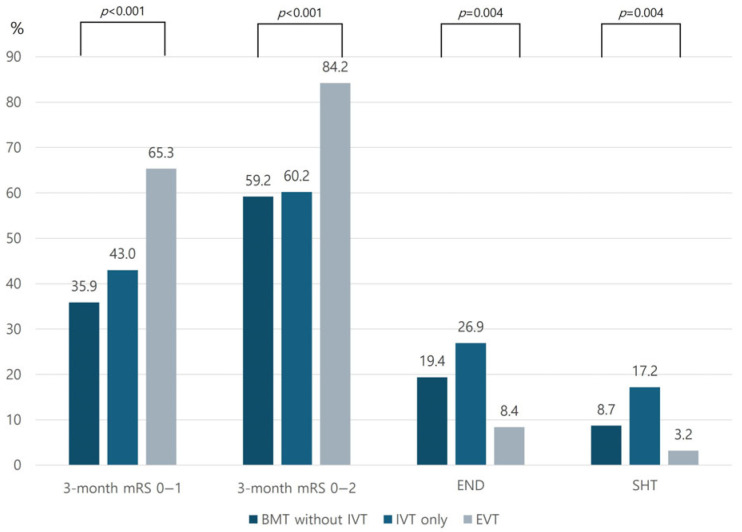
Comparison of stroke outcomes between BMT without IVT, IVT only, and EVT groups. Abbreviations: BMT—best medical treatment; IVT—intravenous thrombolysis; EVT—endovascular treatment; mRS—modified Rankin Scale; END—early neurological deterioration; SHT—symptomatic hemorrhagic transformation.

Compared with the BMT group, the EVT group had a higher rate of an mRS score of 0–1 at 3 months (BMT: 39.3% vs. EVT: 65.3%, *p* < 0.001). In addition, the EVT group had a lower END rate and higher mRS score of 0–2 at 3 months (Figure 2). The safety outcome as a rate of SHT was lower in the EVT group than in the BMT group (3.2% vs. 12.8%, *p* = 0.01). If we analyze the BMT group by dividing it into the IVT-only and medical treatment without IVT groups, the END occurrence and functional outcomes at 3 months were better in the EVT group than in the IVT-only group. SHT demonstrated a higher rate in the IVT-only group than in the EVT and BMT without IVT groups (Figure 3).

**Figure 4:**
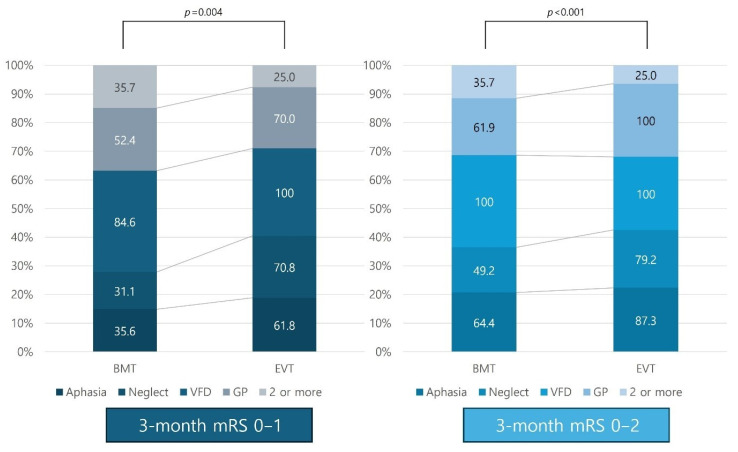
Comparison of 3-month mRS according to cortical signs between BMT and EVT groups. Abbreviations: mRS—modified Rankin Scale; BMT—best medical treatment; EVT—endovascular treatment; VFD—visual field defect; GP—gaze preference.

Considering the cortical signs, all the patients with cortical signs showed a better mRS score of 0–1 at 3 months upon undergoing EVT than BMT. Furthermore, the effect of EVT was maximized when the outcome was expanded to an mRS score of 0–2 at 3 months (Figure 4). Among the cortical signs, patients with aphasia, neglect sign, and gaze preference demonstrated better functional outcome recovery at 3 months when they underwent EVT. In patients with more than two cortical signs, EVT was found to have no significant effect on the functional status at 3 months.

**Figure 5:**
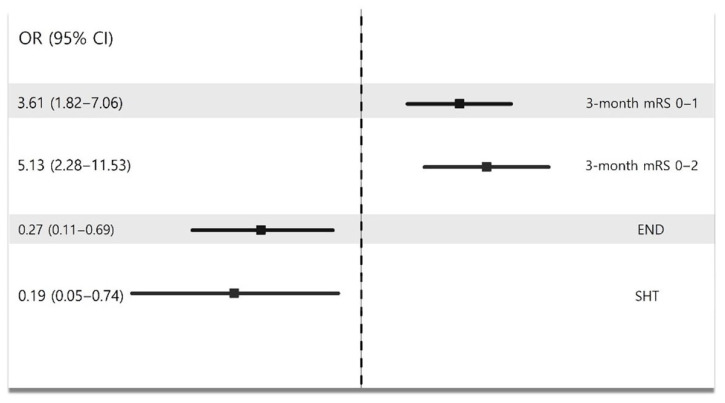
Multivariate analysis showing impact of EVT on stroke outcomes in mild LVO patients.

In the multivariate analysis, EVT was associated with better functional recovery at 3 months (OR [95% CI]: mRS 0–1: 3.61 [1.82–7.06], *p* < 0.001; mRS 0–2: 5.13 [2.28–11.53], *p* < 0.001, Figure 5 and Supplementary Table S1). In addition, EVT could decrease the risk of END occurrence and SHT (END: OR [95% CI], 0.27 [0.11–0.69], *p* = 0.01; SHT: OR [95% CI], 0.19 [0.05–0.74], *p* = 0.02, Figure 5 and Supplementary Table S1).

For the sensitivity analysis, we divided the BMT group into the BMT without IVT and IVT-only groups; EVT demonstrated a 3-month recovery and did not increase the risk of SHT when compared with the impact of IVT only. Patients undergoing IVT only demonstrated an increased likelihood of SHT than those undergoing EVT (Table 2). In addition, when we evaluated the impact of EVT on the 3-month mRS score of 0–1 in the anterior and posterior circulation LVO strokes, EVT was not associated with favorable functional outcome in posterior circulation LVO stroke (Supplementary Table S2).

## 4. Discussion

The results of this study highlight the potential benefit of EVT in patients with LVO stroke with mild symptoms (NIHSS ≤ 5) but prominent cortical signs. Although EVT is the standard treatment for severe strokes with higher NIHSS scores, its efficacy in mild LVO strokes remains controversial, largely owing to concerns about procedural risks, including hemorrhagic complications, in patients with mild initial symptoms. However, our analysis suggests that EVT may indeed improve the functional outcomes in patients with LVO and cortical signs, as evidenced by significantly higher rates of favorable outcomes (mRS score: 0–1) at 3 months than BMT alone.

This study’s findings align with prior observational data, indicating the potential of EVT in specific subgroups of patients with mild LVO stroke, despite limited support from randomized clinical trials for this population [14,15,16,17]. The improvement in functional recovery, coupled with a lower incidence of SHT in the EVT group, supports the notion that carefully selected patients with mild but cortical-sign-presenting LVO may derive a net benefit from EVT. Interestingly, the patients with BMT with IVT-only had higher SHT rate than those with EVT. Although the EVT group included populations who underwent IVT, SHT occurred more frequently in patients with LVO who underwent IVT only. Our study emphasizes that in patients with mild LVO stroke, patients who underwent EVT rather than IVT alone demonstrated improved functional status without increasing bleeding tendency. The EVT group had a higher successful reperfusion rate than the IVT-only group (IVT-only vs. EVT: 36.6% vs. 82.4%, *p* < 0.001). These findings are consistent with previous study findings demonstrating successful EVT reperfusion and improvement of the neurological outcomes at 90 days without increasing the incidence of SHT or mortality in patients with LVO by intra-arterial alteplase administration of Alteplase, which improved neurological outcomes at 90 days without increasing the incidence of SHT or mortality in patients with LVO, and successful reperfusion after EVT [24]. Reperfusion of damaged brain tissue promotes the occurrence of hemorrhagic transformation following IVT, but EVT pre-IVT may help resolve embolism in distal vessels, improve perfusion, and reduce the final volume of damaged brain tissue [25]. Thus, EVT is more effective in improving patient outcomes than IVT alone in patients with LVO who are mildly symptomatic, have cortical symptoms, and arrive within the “golden time window”.

Notably, our study demonstrated that 52% of patients with mild LVO stroke initially had prominent cortical signs. Although this study was not based on a large database, most patients with mild LVO and prominent cortical signs do not undergo EVT in real-world practice. Our findings revealed that cortical signs, such as aphasia, neglect, and gaze preference, were associated with significantly better outcomes when EVT was performed. The residual aphasia and neglect sign had a poor prognosis following stroke [19,26]. This study demonstrated the diminished efficacy of EVT in patients with VFD, which can be attributed to several factors. VFD often arises from ischemia in the occipital lobe, typically due to posterior circulation strokes or distal MCA occlusions. As the occipital cortex does not directly influence motor control or self-care, improvements in VFD may not significantly impact functional outcomes such as mobility or independence measured by the mRS. A previous study demonstrated that patients with occipital infarction had lower mRS and better activities of daily living function [27]. The sensitivity analysis findings evaluating the impact of EVT on the 3-month mRS scores in mild posterior circulation LVO could further support such outcomes. While a previous study with a large cohort demonstrated that EVT improved the functional outcomes [28], recent randomized clinical trials and meta-analysis failed to demonstrate the therapeutic benefit of EVT in posterior LVO stroke [29,30,31]. The limited benefit of EVT in these cases may stem from several factors, including poorer collateral circulation, small brainstem lesion volume leading to high disability, and challenges in early imaging detection. Moreover, cognitive and brainstem-related symptoms may be underestimated by the mRS, which predominantly reflects motor and ambulatory function. Therefore, research on the outcomes of EVT in patients with mild posterior circulation LVO stroke is warranted in the future. In addition, the posterior cerebral artery or distal MCA territories responsible for VFDs are more distal and have smaller-caliber vessels than proximal segments. EVT, while effective for LVOs, may not fully resolve emboli or achieve complete reperfusion in these distal and smaller vessels, limiting its therapeutic benefit [32,33]. These factors highlight the need for tailored strategies in managing strokes with VFD. The patients with multiple cortical signs demonstrated worse outcomes following EVT in this study. Multiple cortical signs may mean involving multiple sites or ischemic lesions. Patients with more than two cortical signs did not benefit significantly from EVT. This may reflect more extensive or multilobar ischemic injury, which may reduce the potential for functional recovery despite reperfusion. Since few patients with multiple lesions and cortical signs underwent EVT, clinicians presumably judged that the potential benefit of EVT would not be significant despite the low initial NIHSS [18]. Our study may help predict the outcome of EVT according to the cortical sign in patients with mild LVO stroke with prominent cortical signs.

The results also demonstrate the heterogeneity within mild LVO stroke cases. The variation in the functional outcomes based on the cortical signs suggests that the NIHSS alone may not capture the complete clinical picture required for EVT decision-making. Traditional reliance on the NIHSS score as the primary determinant of EVT eligibility may overlook certain patients who, despite low NIHSS scores, are at substantial risk of functional decline due to the cognitive and perceptual impairments associated with cortical signs. Our data support the inclusion of cortical sign assessment complementary to the NIHSS for determining patients with mild LVO stroke who are likely to benefit from EVT. Given the limitations of NIHSS in capturing non-motor cortical deficits, future EVT decision-making may benefit from complementary assessments, such as cortical sign-oriented clinical scores or advanced imaging like CT perfusion or collateral grading. Further studies are warranted to confirm these findings.

While these findings are promising, this study also has some limitations. First, the observational design and potential selection bias inherent in registry-based studies warrant caution when generalizing these results; therefore, further randomized trials focusing on patients with mild LVO stroke with cortical signs to validate the efficacy and safety of EVT in this population are warranted. Second, given the significant variability in the collateral status and stroke progression between the anterior and posterior circulations, future studies should examine the effects of EVT on the outcomes in both circulations independently. Third, cognitive decline, which may also affect the functional outcomes, was not assessed in patients who underwent BMT and EVT. Fourth, the ASPECT score was significantly lower in the EVT group than in the BMT group, we overlooked the selection bias that may have resulted in favorable outcomes by performing EVT only in patients with low NIHSS who benefited from EVT. However, this finding suggested that the discrepancy between the extent of infarction and stroke severity, emphasizing that EVT should not be decided solely based on stroke severity in cases with cortical signs in real-world practice. Fifth, although patients in the EVT group arrived significantly earlier than those in the BMT group, our multivariate analysis adjusted for this difference, and EVT remained independently associated with favorable outcomes. Furthermore, the BMT group presented within approximately three hours of symptom onset, which is often considered the “golden window” for reperfusion therapy. The fact that EVT was not performed despite the timely arrival of these patients suggests that factors other than the time from symptom onset to arrival at the hospital, such as an underestimation of stroke severity due to a low NIHSS score, may have contributed to poorer outcomes. Thus, our findings emphasize that timely arrival alone is insufficient; clinical decision-making regarding EVT must also consider cortical signs to avoid missed therapeutic opportunities. Additionally, although we presented crude outcomes by cortical sign type (Figure 4), we did not perform subgroup analyses due to the small number of cases in each category. Our primary aim was to evaluate the overall effectiveness of EVT in patients with cortical signs and low NIHSS scores. Therefore, we did not statistically compare the effectiveness of EVT across specific cortical sign subgroups. Future studies with larger sample sizes are needed to investigate how EVT effectiveness differs based on individual cortical sign presentations. Finally, we did not estimate the initial infarct volume, which limits our ability to correlate lesion burden with the number of cortical signs or outcomes of the entire population in this study. However, our study aimed to evaluate the clinical significance of the initial cortical signs as a surrogate marker of initial stroke severity in EVT decision-making. While assessment of the initial infarct volume may not be possible in emergent settings, initial cortical signs can favor EVT in patients with mild LVO stroke based on our study results. Furthermore, cognitive function, an important factor in cortical strokes, was not systematically evaluated and may have affected recovery trajectories separately from the mRS. Similar studies in the future will benefit from conducting delayed cognitive assessments in addition to mRS to evaluate patient prognosis.

In conclusion, our study suggests that EVT may provide significant functional benefits in patients with mild LVO stroke who present with cortical signs. The presence of these signs may be an important consideration in EVT decision-making, even in patients with initially low NIHSS scores. These findings support a more nuanced approach to the EVT eligibility criteria, potentially contributing to the development of refined clinical guidelines for improving individualized patient care in acute stroke management.

## Figures and Tables

**Table 1:** Baseline characteristics between BMT and EVT groups.

	BMT (n = 196)	EVT (n = 95)	*p*-Value
Age, year (SD)	68.2 (13.8)	65.3 (14.1)	0.60
Male, n (%)	115 (58.7)	64 (67.4)	0.16
NIHSS, (IQR)	4 (2–5)	4 (2–5)	0.70
Onset to arrival, hour (IQR)	3.3 (2.2–6.3)	0.7 (0.5–0.9)	<0.001
Stroke mechanism, n (%)			0.93
LAA	71 (36.2)	36 (37.9)	
CE	70 (35.7)	34 (35.8)	
others	55 (28.1)	25 (26.3)	
Prior stroke, n (%)	38 (19.4)	16 (16.8)	0.63
Hypertension, n (%)	127 (64.8)	54 (56.8)	0.20
Diabetes mellitus, n (%)	66 (33.7)	28 (29.5)	0.51
Hyperlipidemina, n (%)	41 (20.9)	19 (20.0)	0.88
Smoking, n (%)	49 (25.0)	21 (22.1)	0.66
Atrial fibrillation, n (%)	72 (36.7)	33 (34.7	0.80
Prior antithrombotics, n (%)	70 (35.7)	33 (34.7)	0.90
Prior statin, n (%)	51 (26.0)	26 (27.4)	0.89
Lesion location, n (%)			0.17
Anterior	120 (61.2)	68 (71.6)	
Posterior	35 (17.9)	15 (15.8)	
Multiple	41 (20.9)	12 (12.6)	
Collateral status, n (%)			0.32
poor	6 (3.1)	2 (2.1)	
intermediate	25 (12.8)	7 (7.4)	
good	165 (84.2)	86 (90.5)	
Cortical signs, n (%)			0.29
Aphasia	87 (44.4)	55 (57.9)	
Neglect	61 (31.1)	24 (25.3)	
GP	13 (6.6)	4 (4.2)	
VFD	21 (10.7)	8 (8.4)	
2 or more	14 (7.1)	4 (4.2)	
ASPECTS, score (IQR)	8 (8–9)	6 (9–10)	<0.001

Abbreviation: BMT—best medical treatment; EVT—endovascular treatment; SD—standard deviation; NIHSS—National Institute Health Stroke scale; IQR—interquartile range; LAA—large artery atherosclerosis; CE—cardioembolism; GP—gaze preference; VFD—visual field defect; ASPECTS—Alberta Stroke Program Early CT Score.

**Table 2:** Multivariate analysis showing impact of IVT only and EVT on stroke outcomes in mild LVO patients.

	3-Month mRS 0–1			3-Month mRS 0–2			END			SHT		
	OR	95% CI	*p*-Value	OR	95% CI	*p*-Value	OR	95% CI	*p*-Value	OR	95% CI	*p*-Value
BMT without IVT	reference			reference			reference			reference		
IVT only	2.13	0.83–5.47	0.12	1.57	0.56–4.35	0.39	2.81	0.91–8.68	0.07	8.48	1.51–47.54	0.02
EVT	7.53	2.37–23.99	0.001	8.13	2.14–30.97	0.002	0.75	0.18–3.14	0.70	1.59	0.19–13.56	0.67
Age	0.97	0.95–0.99	0.004	0.97	0.94–0.99	0.01	1.02	0.99–1.04	0.25	1.003	0.97–1.04	0.84
Male	0.63	0.37–1.09	0.10	1.42	0.78–2.58	0.25	1.47	0.74–2.91	0.27	0.77	0.32–1.56	0.57
Stroke mechanism												
others	reference			reference			reference			reference		
CE	0.72	0.38–1.37	0.32	0.63	0.33–1.23	0.18	0.79	0.36–1.73	0.56	0.37	0.13–1.05	0.06
LAA	1.25	0.65–2.41	0.50	4.24	1.93–9.33	<0.001	0.79	0.36–1.74	0.55	0.43	0.16–1.17	0.09
Initial NIHSS	1.11	0.96–1.30	0.17	1.01	0.85–1.19	0.93	1.08	0.89–1.31	0.45	1.09	0.85–1.40	0.51
Collateral status												
poor	reference			reference			reference			refernce		
intermediate	1.41	0.22–9.02	0.72	0.43	0.07–2.64	0.36	4.40	0.43–44.97	0.21	2.45	0.22–27.33	0.47
good	6.38	1.4–38.99	0.045	3.67	0.58–23.02	0.17	1.89	0.18–19.87	0.60	0.86	0.07–10.83	0.91
ASPECTS	0.46	0.29–0.75	0.002	0.45	0.26–0.77	0.004	1.29	0.72–2.33	0.39	1.31	0.58–2.96	0.51
Interval from onset to arrival	1.01	0.86–1.19	0.92	1.01	0.85–1.20	0.92	1.16	0.96–1.39	0.12	1.29	1.00–1.67	0.048

Abbreviation: IVT—intravenous thrombolysis; BMT—best medical treatment; EVT—endovascular treatment; mRS—modified Rankin Scale; END—early neurologic deterioration; SHT—symptomatic hemorrhagic transformation; OR—odd ratio; CI—confidence interval; LAA—large artery atherosclerosis; CE—cardioembolism; NIHSS—National Institute Health Stroke scale; ASPECTS—Alberta Stroke Program Early CT Score.

## Data Availability

The datasets generated during and/or analyzed during the current study are available from the corresponding author on reasonable request.

## Supplementary Materials

The following supporting information can be downloaded at: https://www.mdpi.com/article/10.3390/biomedicines13071700/s1, Table S1: Multivariate analysis showing impact of EVT on stroke outcomes in mild LVO patients; Table S2: Sensitivity analysis showing the impact of EVT on 3-month mRS 0-1 according to lesion location.
