# Supplementary material for: Effectiveness and Safety of Endovascular Treatment in Large Vessel Occlusion Stroke with an NIHSS Score of ≤5 Exhibiting Predominant Cortical Signs
Source: Biomedicines. 2025 Jul 11;13(7):1700. doi: 10.3390/biomedicines13071700 (PMC12292358; doi:10.3390/biomedicines13071700)
Supplement: Supplementary file 1 [file biomedicines-13-01700-s001.zip › biomedicines-3719029-supplementary.pdf]

Supplementary Table S1. Multivariate analysis showing impact of EVT on stroke outcomes in mild LVO patients.

	3-month mRS 0-1			3-month mRS 0-2			END			SHT		
	OR	95% CI	p-value	OR	95% CI	p-value	OR	95% CI	p-value	OR	95% CI	p-value
EVT	3.61	1.82-7.06	<0.001	5.13	2.28-11.53	<0.001	0.27	0.11-0.69	0.01	0.19	0.05-0.74	0.02
Age	0.97	0.95-0.99	0.002	0.97	0.94-0.99	0.01	1.01	0.99-1.04	0.35	0.998	0.97-1.03	0.92
Male	0.62	0.36-1.07	0.09	1.39	0.77-2.53	0.28	1.41	0.72-2.77	0.32	0.76	0.32-1.81	0.54
Stroke mechanism												
others												
CE	0.72	0.38-1.35	0.3	0.63	0.32-1.21	0.17	0.77	0.36-1.68	0.52	0.38	0.14-1.05	0.06
LAA	1.27	0.66-2.44	0.47	4.26	1.94-9.35	<0.001	0.82	0.37-1.78	0.61	0.48	0.18-1.28	0.15
Initial NIHSS	1.12	0.95-1.30	0.16	1.01	0.85-1.20	0.91	1.08	0.89-1.31	0.44	1.06	0.85-141	0.49
Collateral status												
poor												
intermediate	1.33	0.21-8.47	0.77	0.41	0.07-2.50	0.33	3.67	0.37-36.58	0.27	1.72	0.16-18.57	0.66

good	4.99	0.85-29.32	0.08	3.03	0.51-18.20	0.23	1.31	0.13-13.22	0.82	0.48	0.04-5.70	0.56
ASPECTS	0.52	0.33-0.82	0.01	0.49	0.29-0.81	0.01	1.52	0.87-2.65	0.14	1.74	0.82-9.70	0.15
Interval from onset to arrival	0.93	0.82-1.06	0.3	0.96	0.84-1.11	0.59	1.04	0.90-1.20	0.58	1.04	0.86-1.25	0.69

Abbreviation: EVT, endovascular treatment; mRS, modified Rankin Scale; END, early neurologic deterioration; SHT, symptomatic hemorrhagic transformation; OR, odd ratio; CI, confidence interval; LAA, large artery atherosclerosis; CE, cardioembolism; NIHSS, National Institute Health Stroke scale; ASPECTS, Alberta Stroke Program Early CT Score

Supplementary Table S2. Sensitivity analysis showing the impact of EVT on 3-month mRS 0-1 according to lesion location.

Anterior circulation				Posterior circulation			
	OR	95% CI	p-value		OR	95% CI	p-value
EVT	5.18	2.23-12.04	<0.001	EVT	1.56	0.28-8.58	0.61
Age	0.99	0.96-1.01	0.33	Age	0.92	0.86-0.98	0.01
Male	0.51	0.25-1.02	0.06	Male	0.61	0.12-3.27	0.57
Stroke mechanism				Stroke mechanism			
Others				Others			
CE	1.01	0.44-2.32	0.99	CE	1.13	0.21-6.13	0.89
LAA	1.42	0.61-3.30	0.41	LAA	0.62	0.10-3.63	0.59
Initial NIHSS	1.2	0.98-1.47	0.07	Initial NIHSS	1.06	0.66-1.70	0.81
Collateral status*	2.19	0.94-5.08	0.07	ASPECTS			
ASPECTS	0.7	0.41-1.18	0.18	Time interval from onset to arrival	0.29	0.08-1.07	0.06
Time interval from onset to arrival	1.02	0.88-1.20	0.76				

Abbreviation: EVT, endovascular treatment; mRS, modified Rankin Scale; END, early neurologic deterioration; SHT, symptomatic hemorrhagic transformation; OR, odd ratio; CI, confidence interval; LAA, large artery atherosclerosis; CE, cardioembolism; NIHSS, National Institute Health Stroke scale; ASPECTS, Alberta Stroke Program Early CT Score

* Collateral status was not adjusted when performing multivariate analysis using the cohort of posterior circulation LVO because only 1 patient had poor collateral status.
